# Genetic stability and phytochemical analysis of the *in vitro* regenerated plants of *Dendrobium nobile* Lindl., an endangered medicinal orchid

**DOI:** 10.1016/j.mgene.2014.06.003

**Published:** 2014-07-15

**Authors:** Paromik Bhattacharyya, Suman Kumaria, Reemavareen Diengdoh, Pramod Tandon

**Affiliations:** Plant Biotechnology Laboratory, Department of Botany, Centre for Advanced Studies, North-Eastern Hill University, Shillong 793022, Meghalaya, India

**Keywords:** *In vitro* propagation, Genetic fidelity, RAPD, SCoT, Antioxidants, Secondary metabolites

## Abstract

An efficient genetically stable regeneration protocol with increased phytochemical production has been established for *Dendrobium nobile*, a highly prized orchid for its economic and medicinal importance. Protocorm like bodies (PLBs) were induced from the pseudostem segments using thidiazuron (TDZ; 1.5 mg/l), by-passing the conventional auxin–cytokinin complement approach for plant regeneration. Although, PLB induction was observed at higher concentrations of TDZ, plantlet regeneration from those PLBs was affected adversely. The best rooting (5.41 roots/shoot) was achieved in MS medium with 1.5 mg/l TDZ and 0.25% activated charcoal. Plantlets were successfully transferred to a greenhouse with a survival rate of 84.3%, exhibiting normal development. Genetic stability of the regenerated plants was investigated using randomly amplified polymorphic DNA (RAPD) and start codon targeted (SCoT) polymorphism markers which detected 97% of genetic fidelity among the regenerants. The *PIC* values of RAPD and SCoT primers were recorded to be 0.92 and 0.76 and their *R_p_* values ranged between 3.66 and 10, and 4 and 12 respectively. The amplification products of the regenerated plants showed similar banding patterns to that of the mother plant thus demonstrating the homogeneity of the micropropagated plants. A comparative phytochemical analysis among the mother and the micropropagated plants showed a higher yield of secondary metabolites. The regeneration protocol developed in this study provides a basis for *ex-situ* germplasm conservation and also harnesses the various secondary metabolite compounds of medicinal importance present in *D. nobile*.

## Highlights

• An efficient micropropagation protocol for *Dendrobium nobile*, has been developed.
• Genetic stability evaluated by ScoT and RAPD markers.
• Higher antioxidant activity within the micropropagated plants over the mother plant.
• Efficiency of TDZ as a potent growth regulator.

## Introduction

**Fig. S1:**
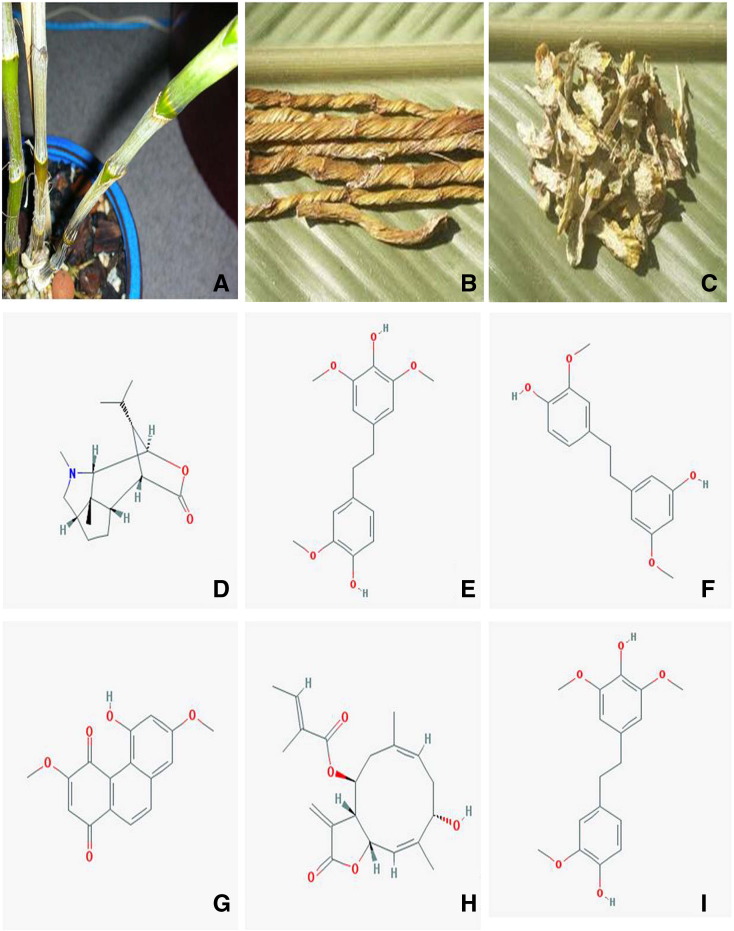
(A) Living stem parts of *D. nobile*. (B) Dried stems of *D. nobile*. (C) Smashed and grinded parts of *D. nobile* and chemical structures of bioactive compounds found from *D. nobile* extracts. (D) Dendrobine. (E) Moscatilin. (F) Gigantol. (G) Denbinobine. (H) Nobilin. (I) Dendrophenol.

Orchidaceae forms one of the world's largest families of flowering plants of angiosperms. Orchids are outstanding in many ways as they have diverse shapes, forms and colors. Orchids are marketed as potted plants and cut flowers leading to their tremendous production over years (Tokuhara and Mii, 2003). However, the orchids in natural habitats are quickly diminishing due to heavy deforestation, urbanization, utilization of land for agriculture and over-exploitation of agro-resources for commercial purposes. To save the diverse orchid species from extinction, *in vitro* culture techniques have been utilized to propagate them (Fay, 1992). Among the various orchids, *Dendrobium nobile* is one of the most commercially and ethno-botanically important taxa. Its violet colored attractive flowers and pattern of flowering have made *D. nobile* a major stake holder in the worldwide cut flower market (Martin and Madassery, 2006). In addition, it has a great use traditionally in various herbal drug preparations (Ye et al., 2002). The presence of various active compounds like dendrobine, moscatilin, gigantol, denbinobine, nobiline and dendrophenol in the stems and leaves of *D. nobile* has greatly increased its medicinal importance (Fig. S1) (Miyazawa et al., 1997, Suzuki et al., 1973, Zhao et al., 2001). These compounds have strong antimutagenic properties and have been found to be anti-carcinogenic against lung carcinoma, ovary adenocarcinoma and promyelocytic leukemia (Lee et al., 1995). Moreover genetic diversity studies have revealed that *D. nobile* from Northeast India has a comparatively high rate of genetic diversity (Bhattacharyya et al., 2013).

Dendrobes are generally propagated asexually by the division of off-shoots, but the multiplication rate is extremely low with only 2–4 plants being produced per year (Martin and Madassery, 2006, Nasiruddin et al., 2003). Thus, conventional methods of propagation are not suitable for large production of high-quality planting material. Like other orchids, the seeds of dendrobes lack endosperm and nutritive substances; hence they are generally difficult to grow into complete plants in nature because seeds require a symbiotic fungus association (Anjum et al., 2006). Thus, to cater the needs of conservation several micropropagation protocols have been successfully developed for various important dendrobes such as *Dendrobium longicornu* (Dohling et al., 2012), *Dendrobium candidum* (Zhao et al., 2008), *Dendrobium densiflorum* (Luo et al., 2008), *Dendrobium lituiflorum* (Das et al., 2008), *Dendrobium chrysotoxum* (Roy et al., 2007) and *Dendrobium transparens* (Sunitibala and Kishor, 2009).

*In vitro* propagation of *D. nobile* has been reported by two workers previously. Nayak et al. (2002) used thin cross sections of *D. nobile* protocorm-like bodies (PLBs) as explants and 6-benzyl amino purine (BAP) and indole-6 butyric acid (IBA) as PGRs for micropropagation. Malabadi et al. (2005) used shoot tips as explants and triacontanol (TRIA) for shoot induction and proliferation. On the other hand, although Wang et al. (2009) reported the use of thidiazuron (TDZ) on *in vitro* flowering of *D. nobile*, the impact of TDZ on the micropropagation of this genus is lesser known. TDZ (N-phenyl-N01,2,3-thiadiazol-5-yl urea; DROPP), primarily used as a cotton defoliant, proved to possess a strong cytokinin-like activity similar to that of adenine derivatives (Mok et al., 1987). Initially TDZ was classified as a cytokinin (Murthy et al., 1998); later researches showed that unlike conventional cytokinins it is capable of supplementing both the cytokinin and auxin requirements of various regenerative plant responses. The exact pathway of TDZ functioning is not known, however, it is assumed that TDZ gets absorbed mainly through leaves. The prominent responses of TDZ treatment in various species include efficient seed germination, expedited bud break, induction and stimulation of sprouting, cotyledon growth and development, formation of trichomes and stomata appearance on floral parts, and cluster and berry weight of grapes (Guo et al., 2011). Mok and Mok (1985) advocated the possibility of TDZ acting as a direct promoter of growth activities similar to N^6^-substituted cytokinins, or it may induce synthesis and (or) accumulation of endogenous cytokinins. In *Vanda coerulea* and *Cymbidium giganteum*, the efficiencies of TDZ in micropropagation have been well established (Malabadi et al., 2004, Roy et al., 2012).

Besides the production of quality plant material, plant cell and tissue culture methods are currently being used in secondary metabolite production (Rout et al., 2000, Verpoorte et al., 2002, Yesil-Celiktas et al., 2007), plant cell biosynthetic capabilities for obtaining useful product (Verpoorte et al., 2002), selection of high metabolite production cell lines (Furmanowa et al., 1998) and studying the metabolism (Misawa, 1994). Various chemical compounds are found in these medicinal plants, of which polyphenols have received much attention because of their role in several degenerative and aging related diseases (Brewer, 2011, Procházková et al., 2011). It has been reported that polyphenols exhibit powerful antioxidant activity in different *in vitro* cellular models and have been consistently protective through scavenging diverse reactive oxygen species (ROS) including hydroxyl radical, peroxyl radical, hypochlorous acid, superoxide anion and peroxynitrite (Halliwell, 2008).

Clonal fidelity of *in vitro* regenerated plants is another aspect of plant propagation which is imperative for their commercial utilization. In spite of various advantages of the *in vitro* propagation, genetic instability has been observed in tissue culture-derived plants (Larkin and Scowcroft, 1981). Clonal stability can be assessed by studying chromosome numbers, isozyme profile and PCR-based molecular markers like random amplified polymorphic DNA (RAPD), inter simple sequence repeats (ISSR) and most recently start codon targeted (SCoT) (Devi et al., 2013, Rathore et al., 2014). Molecular techniques are more advantageous over other methods because they are not influenced by environmental factors and generate reliable and reproducible results. Out of various molecular markers used for evaluation of genetic fidelity of *in vitro* regenerated plants, RAPD is one of the most simple, quick and cost-effective methods (Lakshmanan et al., 2007). But in spite of the various advantages, RAPD has a major issue with reproducibility. SCoT, on the other hand, is extremely reliable and consistent. The SCoT primers have been designed in accordance with the short conserved region surrounding the ATG translation start (or initiation) codon (or translational start site, TSS). More specifically, it is a type of targeted molecular marker technique with the ATG context as one part of a functional gene; markers generated from SCoT may be mostly correlated to functional genes and their corresponding traits (Bhattacharyya et al., 2013, Collard and Mackill, 2009). Various assays have been carried out to analyze the reported mutagenic effects of TDZ on the *in vitro* propagated plantlets and subsequent development of somaclonal variations. Ferreira et al. (2006) found no variations in RAPD profiles among the *in vitro* generated plantlets of *Dendrobium* (Second Love) derived through TDZ treatment. Likewise, no variation was observed due to TDZ treatment among the *in vitro*-derived orchid plantlets. But, Chen et al. (1998) and Khoddamzadeh (2010) found variability in RAPD profiles among the *in vitro*-derived plantlets of *Phalaenopsis bellina*. However, only few investigations exist on genetic fidelity of the *in vitro*-regenerated orchids with special reference to dendrobes.

In order to formulate a more synergistic and effective propagation strategy for this medicinally important endangered orchid taxa which is facing threats of extinction due to anthropogenic pressures and habitat destruction, we describe here the efficiency of TDZ on high frequency micropropagation of *D. nobile* from pseudostem segments. Genetic variability within the propagated plants was also analyzed using RAPD and SCoT markers to determine the efficacy of the protocol from the conservation aspects. Biochemical parameters and their yields *viz.* total phenolic content, total flavonoid content and antioxidant activity were investigated and compared between the mother and TDZ induced micropropagated plants.

## Materials and methods

### Explant source and culture conditions

**Fig. 1:**
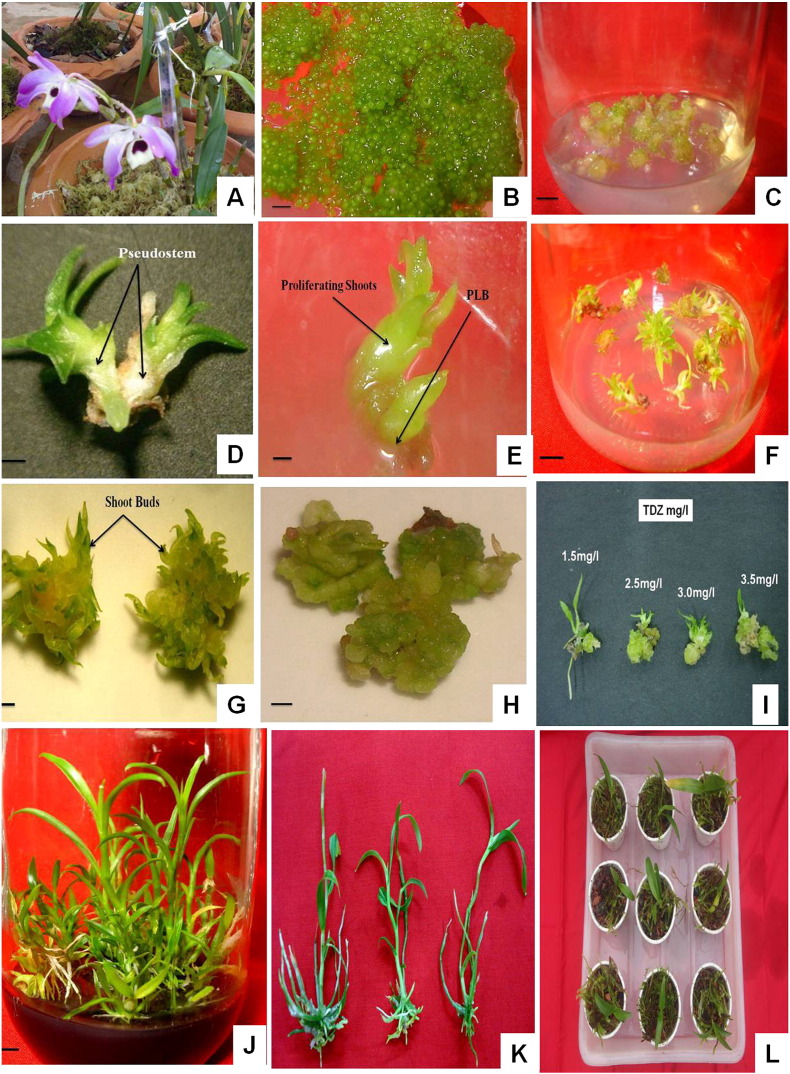
Micropropagation of *D. nobile* from pseudostem segments: (A) mature plant in the greenhouse, (B) asymbiotic seed germination in MS medium, (C) protocorm development into plantlets in MS medium (bar = 1 cm), (D) pseudostem segments excised as explants with two leaves (bar = 1 cm), (E) initiation of shoot proliferation from pseudostem explants (bar = 1 cm), (F) PLBs induced from explants cultured in MS medium + 1.5 mg/l TDZ (bar = 1 cm), (G) multiple shoots originating from induced PLBs + 1.5 mg/L TDZ, (H) compact mass of PLBs formed when exposed to 3.0 and 3.5 mg/l TDZ, (I) differential responses of PLBs to concentrations of TDZ, (J) complete plantlet with roots after 8 weeks of culture, (K) completely rooted plantlets after 8 weeks of culture, and (L) greenhouse acclimatized plantlets.

The plants of *D. nobile* were collected from Pakyong (Sikkim) and were maintained in the greenhouse of the Plant Biotechnology Laboratory, Department of Botany, North-Eastern Hill University, Shillong, India (Fig. 1A). About 8–9 month old green capsules of *D. nobile* were collected and washed thoroughly under running tap, then treated with 10% Teepol (Qualigens Fine Chemicals, Mumbai, India), surface sterilized using 0.04% (w/v) Bavistin solution (BASF, Mumbai, India) for 20 min, and washed thrice with sterile millipore water. Finally, the capsules were dipped in 70% ethanol for 30 s followed by flaming for 2–3 s. The sterilized capsules were excised longitudinally with a sterile surgical blade and around 1 g of seeds was inoculated on Murashige and Skoog medium (MS, 1962) for germination and plant development (Murashige and Skoog, 1962). Pseudostem segments with nodes (0.5–1 cm) cut from the base of *in vitro*-raised seedlings with two fully expanded leaves were used as explants.

### PLB inductions from pseudostem segments

Pseudostem segments were cultured in combinations of eight different concentrations (0.1, 0.5, 1.0, 1.5, 2.0, 2.5, 3.0, 3.5 mg/l) of TDZ for induction of PLBs.

### Plantlet development and transfer

Complete *in vitro* regenerated plantlets of *D. nobile* measuring about 2.5–3.0 cm in height were transferred to pots with different potting mixtures constituted of brick, charcoal and decaying litters *viz.*, (1) charcoal pieces and bricks (1:1), (2) decaying litter and brick pieces (1:1), (3) brick chips, decaying leaf litter and charcoal pieces (1:1:1), and (4) brick chips, decaying leaf litter and charcoal pieces (1:1:1) along with a top layer of moss. The glass house temperature used during hardening was maintained at 25 ± 2 °C along with a relative humidity level of 70–80%. The plantlets were watered daily and fed with MS nutrient salt solutions (10 times diluted) fortnightly for about a month. The experiments were repeated three times with ten replicates per treatment. Statistical analysis was done by analysis of variance (ANOVA) at p < 0.05 and means compared using Fischer's LSD test (PC version Origin 7.0, Northampton, MA, USA).

### Genetic variation analysis of the regenerated plantlets

Total genomic DNA was extracted from young leaves of mother and *in vitro*-propagated *D. nobile* plants following the method described by Murray and Thomson (1980) with some minor modifications. The quantity and purity of the isolated DNA were checked using a UV spectrophotometer (Perkin-Elmer Lambda 35). The ratio of absorbance at two wavelengths (A_260_ and A_280_) was compared with the standard ratio of pure DNA. The quantities of the DNA isolated were found to be optimum for further PCR amplification.

All the PCR reactions containing 40 ng of template DNA, 2 μM each of the four dNTPs, 1 × PCR buffer (10 mM Tris pH 9.0, 50 mM KCl), 2.5 mM of MgCl_2_, 1 U of Taq polymerase (Bangalore Genei, India) and 20 pmol of primer, were carried out in 25 μl volumes of 0.2 ml microfuge tubes (Dialabs). The reaction programs for RAPD were set at 94 °C for 3 min followed by 45 cycles of 94 °C for 45 s, 36 °C for 30 s, 36 °C for 45 s, and 72 °C for 5 min (Kumar et al., 2013) in a Veritti Thermal Cycler (Applied Biosystems, USA), and for SCoT at 94 °C for 4 min, followed by 35 cycles of 30 s at 94 °C, 1 min at 50 °C and 2 min at 72 °C, with a final extension at 72 °C for 5 min for SCoT (Bhattacharyya et al., 2013). After completion of the amplification, 2.5 μl of 10 × blue dye was added to the samples, and the amplified DNA was analyzed on 1.8% agarose gel in 1 × TAE buffer at 65–70 V for 3–4 h. DNA fragments were visualized under UV light and photographed using the Gel Documentation System (Biostep DH-20, Germany).

### Data analysis

Banding profiles generated by RAPD and SCoT primers were pooled into a data binary matrix based on the presence (1) or absence (0) of the selected band. Only clear, unambiguous, and reproducible bands amplified in both cases were considered for the scoring of the data. Smeared and weak bands were excluded. The ability of the primers to distinguish between individuals was accessed by calculating the resolving power (*R_p_*) (Prevost and Wilkinson, 1999) for both the markers. The function is deduced by the formula *R_p_* = Σ*I_b_* where band informativeness, *I_b_* = 1 − (2 × |0.5 − *p*|) and *p* is the proportion of individuals containing band *I*. Polymorphic information content (*PIC*) value was calculated using the formula *PIC* = *1* − Σ*pi*^2^, where *pi* is the frequency of the *i*th allele (Smith et al., 1997). The fit between the similarity matrix and the dendrogram was estimated from the cophenetic correlation coefficient (r) (Sneath and Sokal, 1973). Genetic similarity based on Jaccard's coefficient was calculated using SimQual module and arranged into a similarity matrix. A dendrogram was constructed using a NTSYS version 2.20 software package (Rohlf and Taxonomy, 1998) by following the UPGMA option of the SAHN module.

### Evaluation of secondary metabolites

#### Plant material and extract preparation

Fresh plant parts (stem and leaf) of mother as well as *in vitro* raised plants were washed under running tap water and blotted with tissue towel. The tissue was dried in room temperature. 100 mg of dried tissue was homogenized in 100 ml of respective solvent (methanol, chloroform, and acetone) and extractions were carried in an orbital shaker (REMI, India) with constant stirring at 150 rpm for 24 h. The mixtures were centrifuged at 10,000 rpm for 10 min and the supernatant was filtered through Whatman filter paper (No. 1). Measurements of biochemical parameters were taken on a Lambda-35 double beam spectrophotometer (Perkin-Elmer, USA).

#### Determination of total phenolic content (TPC)

The estimation of total phenolic content was performed by using the Folin–Ciocalteu method (Singleton and Rossi, 1965) with minor modification. Briefly, 0.125 ml of extract was mixed with 1.8 ml of Folin–Ciocalteu reagent (ten times diluted) and kept for 6 min at 25 °C. Then 1.2 ml of 20% Na_2_CO_3_ was added to the reaction mixture and kept for 1 1/2 h at room temperature. The absorbance of the reaction was measured at 765 nm. The concentration of the total phenolics was determined as mg of gallic acid equivalents (GAE) per gram of tissue using an equation obtained from the gallic acid calibration curve.

#### Determination of total flavonoid content (TFC)

Total flavonoid content was determined by using the aluminium chloride colorimetric method with minor modifications (Chang et al., 2002). Briefly, 0.5 ml of extracts, 1.5 ml of methanol, 0.1 ml of aluminium chloride (10%), 0.1 ml of sodium acetate (1 M) and 2.8 ml of distilled water were mixed for 5 min by vortexing. The reaction mixture was kept at room temperature for 30 min and the absorbance was measured at 415 nm. The calibration curve was prepared for quercetin and the results are expressed as mg of quercetin equivalents (QE) per gram of tissue.

#### Determination of total tannin content (TTC)

Total tannin content was measured by using the Folin–Dennis method (Singleton et al., 1999). 0.25 ml of extracts was mixed with 0.5 ml of Folin–Dennis reagent. 1 ml of 20% sodium carbonate solution and 1 ml of millipore water was added thereafter. The reaction mixture was incubated at room temperature for 30 min. The absorbance of the reaction was measured at 775 nm. The concentration of the total tannin was determined as mg of tannic acid equivalents (TAE) per gram of tissue using an equation obtained from the tannic acid calibration curve.

#### Determination of total alkaloid content (TAC)

The estimation of total alkaloid content was performed by using Dragendorff's alkaloid estimation method with minor modifications (Sreevidya and Mehrotra, 2003). Dragendorff's reagent was prepared by mixing 1.7 g of Bismuth subnitrate, 20 ml of glacial acetic acid, 80 ml of millipore water and 50% solution of potassium iodide (50 g in 100 ml). Out of this Dragendorff's stock solution, 10 ml was missed with 20 ml of glacial acetic acid and the final volume was constituted up to 100 ml with millipore water. This solution was used as the working solution for the further experiments. 0.25 ml of the plant extract was then mixed with 0.5 ml of Dragendorff's reagent working solution and was kept at room temperature for 45 min. The absorbance of the reaction was measured at 700 nm. The concentration of the total alkaloid was determined as mg of atropine equivalent (AE) per gram of tissue using an equation obtained from the atropine calibration curve.

#### Antioxidant activity of micropropagated plants

##### DPPH free radical scavenging assay

The DPPH (2,2-diphenyl-1-picrylhydrazyl) free radical scavenging activity of the extracts was determined essentially as described by Brand-Williams et al. (1995) and modified by Jagtap et al. (2011). The stock reagent solution was prepared by dissolving 24 mg of DPPH in 100 ml methanol and stored at − 20 °C until use. 100 μl of extract was allowed to react with the DPPH solution in the final reaction volume of 3 ml. The mixture was shaken vigorously and allowed to stand in the dark at room temperature. The decrease in absorbance of the resulting solution was then measured spectrophotometrically at 517 nm. The control was prepared as above without any extract and MeOH was used for the baseline correction. Radical scavenging activity was expressed as the inhibition percentage and was calculated using the following formula:

$$\%\;\mathrm{Radical}\;\mathrm{Scavenging}\;\mathrm{Activity}\;\left(\mathrm{Brand}‐\mathrm{Williams}\;\mathrm{et}\;\mathrm{al}.,\;1995\right)=\left(\mathrm{Control}\;\mathrm{OD}-\mathrm{Sample}\;\mathrm{OD}/\mathrm{Control}\;\mathrm{OD}\right)\times 100.$$

##### Ferric Chloride Reducing Power (FRAP) assay

The FRAP assay was performed using the methodology described by Benzie and Strain (1996). The working FRAP reagent was prepared by mixing 0.3 M acetate buffer (pH 3.6), 10 mM 2,4,6-tripyridyl-s-triazine (TPTZ) in 40 mM HCl and 20 mM FeCl_3_, 6 H_2_O in 10:1:1 ratio prior to use and heated at 37 °C in water bath for 10 min. 100 μl of extract was used for the reaction with 2.7 ml of the FRAP reagent. The final reaction volume was made up to 3 ml with sterile distilled water. The reaction mixture was incubated in the dark for a 30 minute time. The absorbance of the colored complex (ferrous tripyridyltriazine complex) was recorded at 593 nm. An increased absorbance power indicated a higher reducing power.

## Results and discussion

The seeds of *D. nobile* germinated within 7 weeks of culture in nutrient medium. The protocorms differentiated into seedlings after 4 weeks of germination (Fig. 1B, C). Clonal propagation through PLBs or shoot buds from different explants of dendrobes has been used for the propagation protocols. In such cases, PLB induction under controlled conditions has been used to study the influence of abiotic and biotic factors (Teixeira da Silva et al., 2006), medium constituents, carbon source, PGR, *etc.* (Kishor and Devi, 2009, Nasiruddin et al., 2003). In the present study, TDZ incorporated in MS medium was used for PLB induction and subsequent organogenesis in the segments of pseudostem explants (Table 1). Depending upon the concentrations of TDZ in the medium, the pseudostem explants produced compact masses of green PLBs. Initially, small projections resulted from the explants within 4 weeks of culture which proliferated eventually into multiple shoots and plantlets within 8 weeks of the culture (Fig. 1D–G). The highest explant response was 94.1% at 1.5 mg/l of TDZ. The synergistic effect of TDZ, in the present study, has been observed for efficient PLB induction from *D. nobile* explants so as to detect the immediate and long term effects of TDZ on the clonally propagated plants of this orchid species.

In most of the orchids, cytokines either singly or in combination with auxins have been shown to induce PLBs (Bhattacharyya et al., 2013, Dohling et al., 2012, Miyazawa et al., 1997, Nasiruddin et al., 2003, Zhao et al., 2008). The present study reconfirms the efficiency of TDZ as a substitute for auxin–cytokinin combination (Chen et al., 1998, Kishor and Devi, 2009, Malabadi et al., 2005, Mok and Mok, 1985) and proves TDZ to be better for the propagation of *D. nobile* than the conventional auxin–cytokinin complement used (Asghar et al., 2011, Nayak et al., 2002). Although the present study revealed a higher number of PLBs' induction at higher TDZ concentration, it was found that the conversion rate of shoot regeneration from these PLBs got restricted. It was also observed that at lower TDZ concentrations in the medium (0.5 and 1.0 mg/l) the rate of PLB induction per explant was low (5.77 and 7.50). However, the PLBs developed were much healthier with several proliferating shoots than those induced at higher concentrations of TDZ, and some of them simultaneously differentiated into shoots and roots (Fig. 1F, G). Culture weight, number of shoots per explant, shoot length and root length were also significantly better at lower to moderate concentrations of TDZ (Table 1). Comparatively, at 2.5 mg/l TDZ in the medium, high number of PLBs (12.80/explant) was induced but the cultures developed a combination of compact mass of PLBs and the shoots that differentiated were less in height and were of stunted growth (Fig. 1H). Chang and Chang (2000) also reported optimum shoot bud induction and organogenesis at lower concentrations of TDZ and complete inhibition of rhizome induction at higher concentrations in the case of *Cymbidium sinense*. Also, in *D. chrysotoxum*, TDZ at lower concentration with NAA was found to be most responsive in inducing direct PLB formation (Roy et al., 2007). Similar effectiveness of low TDZ concentration on shoot proliferation and organogenesis has also been reported in orchids (Malabadi et al., 2004, Roy et al., 2012) as well as other plants (Bidabadi et al., 2010, Briggs et al., 1987, Jones et al., 2007, Kumar et al., 2010). The probable causes of such results could be related to the (a) induction of synthesis, and (or) accumulation of endogenous cytokinins (Mok and Mok, 1985), (b) concentration specific balancing in the ratio of exogenous PGRs (Tao et al., 2011), (c) nutrients that allow specific modes of regeneration (Jones et al., 2007), and (d) isopentyl adenine mediated rapid cell division and stimulation of shoot organogenesis (Guo et al., 2011). The exposure of the explants to TDZ has been reported to influence shoot proliferation and embryogenesis (Tao et al., 2011). In some plant species, brief exposure ranging from 6 to 20 days, depending on concentration, has been found to be beneficial for shoot proliferation (Liu et al., 2003, Tang and Newton, 2005) whereas in others a prolonged TDZ exposure led to an inhibitory response (Guo et al., 2011). Being a urea based cytokine, TDZ does not get degraded by a cytokinin oxidase enzyme and thus is subjected to residual effects. There are reports suggesting that TDZ has a residual or carry-over effect which helps shoots to proliferate in a hormone-free environment (Makara et al., 2012). The exposure time was also kept sufficiently high (8 weeks) to reduce the chances of residual toxicity.

During micropropagation the release of phenolics is very common and often adversely affects the response of the explants. We wanted to develop a protocol which will lead to the production of increased phenolic and other secondary metabolites in the cultured biomass corroborated with healthy proliferation of plant tissues. Incorporation of activated charcoal has been found to be an efficient adsorbant reducing the adverse phenomenon of tissue browning (Fridborg et al., 1978, Van Waes and Debergh, 1986, Weatherhead et al., 1986). The plantlets responded best for rooting in the medium containing 1 mg/l TDZ in combination with 0.25% activated charcoal (Table S1, Fig. 1I, J, K). These are in agreement with the findings in *Aristolochia indica* (Manjula et al., 1997) and *Dendrobium hookerianum* (Paul et al., 2011). The beneficial effect of activated charcoal is further demonstrated in many plants such as *Annona squamosa* (Lemos and Blake, 1996), *Cymbidium mastersii* (Mohanty et al., 2012) and pineapple (Firoozabady and Gutterson, 2003).

The plantlets were grown in the compost mixture comprising of brick, charcoal, and decaying litter along with a top layer of moss. The highest survivability percentage recorded was 84.3% as compared to other potting mixtures used (Table S2, Fig. 1L). The possible reason behind such a result might be due to the fact that the structural support provided by the substratum and also the presence of air spaces between the substratum particles facilitated the roots to spread and develop properly. Moreover, the required moisture level was maintained by the top layer of moss. During acclimatization of the plantlets, the original shoots died and new shoots emerged after a certain time period which is supported by the findings of Dutra et al. (2008). Also in the present study, feeding the plantlets fortnightly with ten times diluted MS was found to be beneficial for their growth. Similar findings were observed by Paul et al. (2012) in *D. hookerianum*.

**Fig. 2:**
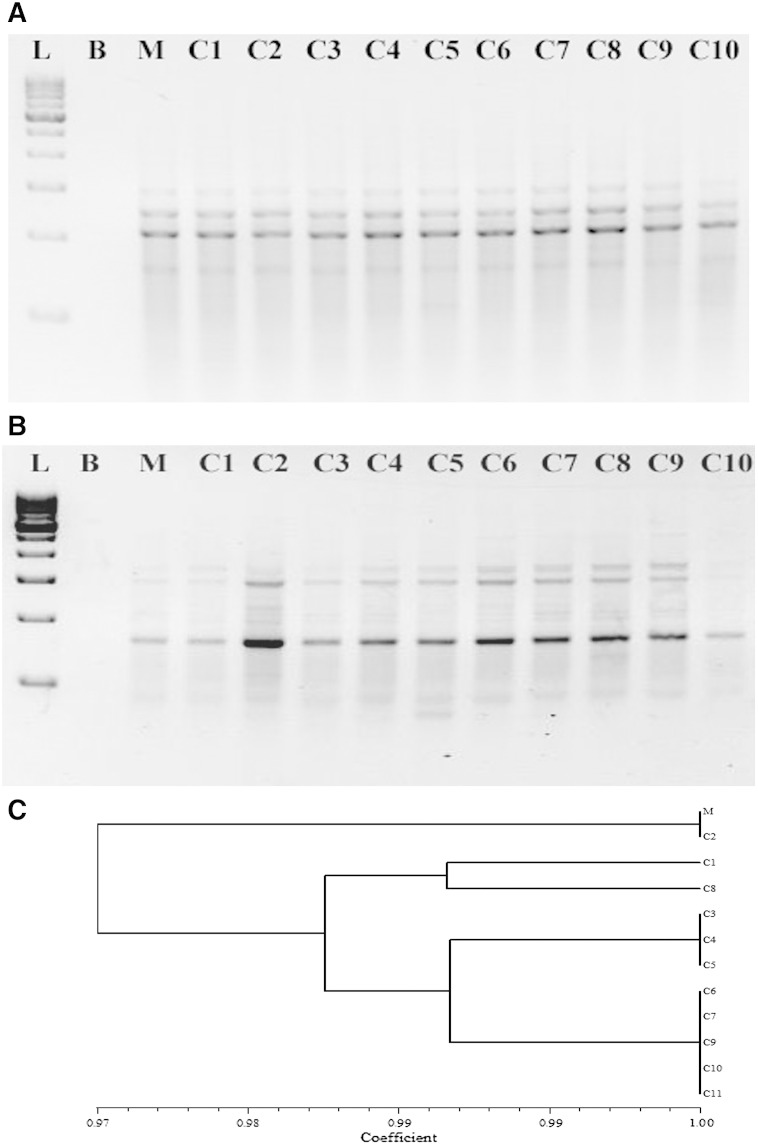
Banding profiles in *D. nobile* using RAPD and SCoT primers. (A) OPA-13 and (B) S34 with lane L — 100 bp ladder; lane B — blank; lane M — mother plant, lanes C1–C10 — micropropagated plants. (C) Dendrogram illustrating coefficient similarities among regenerated plants and the mother plant by the UPGMA cluster analysis using the NTSYS-PC program.

**Fig. 3:**
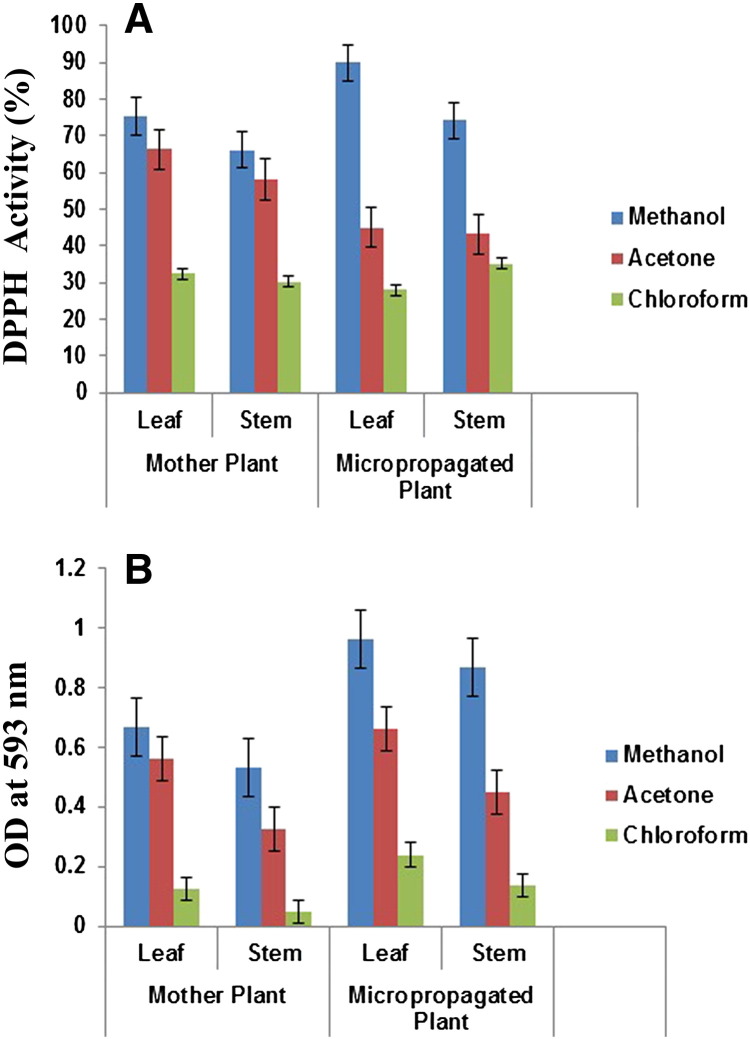
Antioxidant activity of *D. nobile* within the mother and *in vitro* raised plants using methanol, chloroform and acetone as solvents. (A) DPPH activity. (B) FRAP activity.

Studies have shown that plant tissues grown *in vitro* are vulnerable to certain degrees of genetic variations. Under the presence of potent PGR like TDZ and prolonged exposure to it, chances of induction of heritable somaclonal variations within the regenerated plantlets get increased. Furthermore, various reports are there which advocate that explants treated with TDZ develop somaclonal variability within the regenerants (Chen et al., 1998). In commercial as well as conservation programs, such variabilities are proposed to be of much importance compared to that of heritable variations (Roy et al., 2012). In the present study, RAPD and SCoT markers were used to assess the genetic variations in the micropropagated plantlets. A total of 80 RAPD primers were screened; out of which, 13 primers gave reproducible bands. However for assessment of genetic stability of the regenerated plantlets, 7 primers which resulted in clear, unambiguous, consistently reproducible uniform and scorable bands were considered and scored. A total of 27 bands were scored; out of which, only 3 bands were polymorphic, while the rest were monomorphic (Table 3). The number of bands varied from 2 (OPK-4; OPB-1) to 6 (OPA-11) (Fig. 2A; Table 2) and their *R_p_* value ranged from 3.66 (OPB-1) to 10 (OPH-19). The *PIC* value of the RAPD marker system was 0.92 (Table 3). Using SCoT, a total of 35 primers were screened for assessment of the genetic stability of the regenerated plantlets and among them 15 primers resulted in clear, unambiguous, consistently reproducible uniform and scorable bands. A total of 57 bands were scored; out of which, only 2 bands were polymorphic, while the rest were monomorphic (Table 3). The number of bands varied from 4 (S25, S4) to 12 (S5) (Fig. 3B; Table 2) and their *R_p_* value ranged from 4 (S25) to 12 (S5). The *PIC* value of the SCoT marker system was 0.76. The pooled RAPD and SCoT matrix data produced a total of 84 bands, of which 79 fragments were monomorphic (94.04%) with an average of 3.81 bands per primer (Table 3). The *PIC*, correlation and determination coefficients of the pooled marker data were 0.82, 0.51 and 0.27, respectively, subsequently indicating a good fit. The *R_p_* and *PIC* values indicate the efficiency of the primers used and also of the molecular marker used. Both RAPD and SCoT reveal the robustness of the marker systems which is further corroborated by the findings of Bhattacharyya et al. (2013). The degree of variability detected within the propagated plants has been found to be very less (5.95%). However workers have reported higher variability in regeneration protocols using TDZ; RAPD profiling revealed 17% dissimilarity in apple (Modgil et al., 2005). Similarly, Khoddamzadeh (2010) reported the existence of 17% genetic variability in *P. bellina* using RAPD among the regenerants raised from TDZ treatment at 3 mg/l. On the other hand, lesser degree of genetic variability among the regenerated plantlets has been reported in some other orchids propagated through *in vitro* propagation using TDZ as PGR (Ferreira et al., 2006). The percentage of genetic variability recorded in this study was lower than those induced by TDZ in other orchids which is in agreement with the findings of Ferreira et al. (2006). RAPD technique has been extensively used to assess genetic variability generated by *in vitro* techniques (Chen et al., 1998, Devi et al., 2013, Ferreira et al., 2006, Khoddamzadeh, 2010, Roy et al., 2012); however, the successful assessment of RAPD profiles generated requires validation through repeated experiments. Thus, the degree of variability detected by RAPD technique needs to be crosschecked by using another marker system (Goto et al., 1998). In fact, sometimes RAPD fails to reveal changes in the repetitive regions of the genome of some species (Palombi and Damiano, 2002). However, it can be used for rapid evaluation of clonal variability within the micropropagated plants, by random scanning of the whole genome. On the other hand, SCoT markers reveal the genetic diversity at gene levels thus increasing the possibility of finding new alleles among a given germplasm collection making the variations revealed by it much more precise (Collard and Mackill, 2009). The quantification of polymorphic loci is an important parameter used in the genetic fidelity analysis of a population. In the present study, pooled RAPD and SCoT matrix revealed 79 out of 84 bands to be monomorphic (94.04%) whereas 5 bands were polymorphic (5.95%) showing a high degree of genetic stability within the *in vitro* propagated plants. Jaccard's distance matrix ranged from 0.97 to 1.00 revealing a higher degree of genetic relatedness which is also supported by the quantification data of the polymorphic loci and the findings of Roy et al. (2012) in *C. giganteum*. Our findings are further supported by the observations of other workers (Devi et al., 2013, Ferreira et al., 2006, Lakshmanan et al., 2007, Roy et al., 2012). The significance of the present findings in this study is further justified by the obtained *PIC* and *R_p_* values of the primers used in the study which are significant with the optimal *PIC* (Smith et al., 1997) and *R_p_* values (Prevost and Wilkinson, 1999). These two parameters determine the effectiveness of the markers used in the fingerprinting assay and consequently the novelty of the techniques used. This was further justified by the findings in the Mantel test where the value of correlation coefficient between RAPD and SCoT markers was significant (r = 0.51) and the value of determination coefficient was also high (r^2^ = 0.26) signifying that the two markers identically utilize the existing variation of the *D. nobile* genome.

Orchids house a wide array of secondary metabolites in it, but a very small portion of them have been evaluated. Primarily orchid phytochemicals are alkaloids, flavonoids, carotenoids and sterols. However recently, in *Habenaria edgeworthii*, the presence of polyphenols has been shown (Giri et al., 2012). The level of total phenolics, flavonoids, alkaloids and tannins in plants represents major groups of plant constituents which predominantly work as powerful antioxidants or scavenger of free radicals. They play a significant beneficial role in human health and serve as important remedies of various inflammatory disorders, cancer and diabetes which mainly occur due to the deregulation of free radical generation in the cells (Conner and Grisham, 1996). Being one of the most important medicinal orchids, the knowledge of the biochemical constitution of various parts of *D. nobile* is essential. Traditionally stem has been used for various Chinese herbal preparations, but our findings reveal that the leaves also house a wide array of biochemical entities. Variations were observed in the total phenolic and flavonoid contents of different parts *viz.* stem and leaf of the mother plant and the micropropagated plants (Table S3). Methanolic stem extract of the micropropagated plant exhibited the highest phenolic content (41.39 ± 0.1 mg GAE/g DW) whereas, chloroform leaf extract of the donor plant showed the lowest phenolic content (3.25 ± 0.2 mg GAE/g DW). Similar observations were recorded in *Salacia chinensis* (Chavan et al., 2012) which is also a medicinal plant species. The results obtained in the present study revealed that the level of polyphenols and flavonoids in the methanolic extract of stem and leaf of *D. nobile* was considerable, which was significantly higher than that of chloroform and acetone extracts (Table S3). Additionally, it was reported that phenolic compounds are associated with antioxidant activity and play a significant role in stabilizing lipid peroxidation (Beyhan et al., 2010). Flavonoids quench down the free radicals and thereby reduce their levels significantly, thereby up regulating or protecting antioxidant defense. The solubility of flavonoids was significantly affected by the solvent used for extraction and these findings are in accordance with the results obtained for *Asimina tribloba* (Harris and Brannan, 2009) and *S. chinensis* (Chavan et al., 2012). The variation in the phenolic and flavonoid contents in the different parts might be due to the hormonal content, specific metabolic as well as endogenous physiological changes taking place in the plants. In the present study the highest concentration of flavonoid was found in the methanolic leaf extract of the *in-vitro* grown plants (14.39 ± 0.3 mg QE/g DW) whereas the least was found in the chloroform stem extract of the mother plant (0.53 ± 0.1 mg QE/g DW). Our findings reveal that the contents of various secondary metabolites are varying with the solvent system used as well as with the plant parts. Similar variations of phenolic and flavonoid contents within the plant parts were reported in 12 medicinal plants of the families *Asclepiadaceae* and *Periplocaceae* (Surveswaran et al., 2010).

Like the phenolics and flavonoids, alkaloids are also one of the most phytochemicals found within the orchids and especially the dendrobes. Zhang et al. (2003) reviewed 100 phytochemical compounds from 42 *Dendrobium* species, including 32 alkaloids, 6 coumarins, 15 bibenzyls, 4 flouronones, 22 phenanthrenes and 7 sesquiterpenoids. Our studies have revealed potential higher deposition alkaloids in the leaf tissues. Under the *in-vitro* PGR stress condition, the alkaloid content has increased convincingly in comparison with the mother plant. Also, the solvent system has played a significant role. Methanolic extract from the leaf showed the highest concentration of alkaloids whereas the chloroform extract from the stem showed the lowest concentration (Table S3).

Apart from phenolics, alkaloids and flavonoids, tannins are also a very important plant phytochemical. They are widely distributed in almost all plant parts. Tannins show an effective role in protecting the kidneys and shows potential antiviral (Lü et al., 2004), antibacterial (Akiyama et al., 2001) and anti-parasitic (Kolodziej and Kiderlen, 2005) effects. It's believed that tannins isolated from the stem bark of *Myracrodruon urundeuva* are of neuroprotective attributes (Nobre-Junior et al., 2008). Apart from these effects, tannins also have anti-inflammatory and anti-ulcer properties. It has been found in various rodent based experimental models that they show a very strong antioxidant property for possible therapeutic applications (Souza et al., 2007). In orchids, reports of tannin are very few. Very recently, a very preliminary study was conducted in *Dendrobium panduratum*, where tannin activity was reported (Johnson and Janakiraman, 2013). Methanolic stem extract showed the highest amount of tannin deposition (23.22 ± 0.3 mg TAE/g DW), whereas least was found in the chloroform leaf extract of the mother plant (3.11 ± 0.23 mg TAE/g DW) (Table S3). The amount of TTC estimated from the various plant tissues of *D. nobile* is of significant value and justifies the traditional usage of *D. nobile* in treatment of various stomach disorders.

The free radical scavenging activity of the different parts was tested through DPPH method and the results are presented in Fig. 3A. In the DPPH method, the antioxidants react with the stable free radical *i.e.*, 2,2-diphenyl-b-picrylhydrazyl (deep violet color) and convert it to 2,2-diphenyl-b-picrylhydrazine with discoloration. The degree of discoloration indicates the scavenging potentials of the sample antioxidant. In the present study the extracts of different parts were able to decolorize DPPH. Among various solvents and plant parts tested, the methanolic leaf extract of the micropropagated plant showed the highest DPPH activity (89.8 ± 2.9%), while the chloroform leaf extract of the micropropagated plant exhibited the lowest radical scavenging activity (28 ± 2.1%) (Fig. 3A). The methanolic extract obtained from the peel of pineapple (Hossain and Rahman, 2011) and from the fruit pulp of *S. chinensis* (Chavan et al., 2012) exhibited the highest antioxidant activity. The *in vitro* raised plants of *D. nobile* exhibited a higher degree of free radical scavenging activity than the mother plant. Similar findings were observed in *Piper nigrum* (Ahmad et al., 2010) and in *Aloe arborescens* (Amoo et al., 2012) where a higher potential of free radical scavenging activity was recorded. Although variations were detected among the extracts from the various parts of *D. nobile*, the average range of scavenging is over 50% which proves that it possesses hydrogen donating capabilities to act as a potent antioxidant.

Like DPPH, FRAP is also a very commonly used antioxidant assay used in the analysis of antioxidant capacities of medicinal plants. It's a very simple, rapid, sensitive and inexpensive approach. The reducing capacity of a compound might serve as a significant indicator of its antioxidant capacity. The antioxidant capacity of different parts using FRAP assay is shown in Fig. 3B. The extent of reduction in terms of absorbance was observed at 593 nm. The highest ferric reducing capacity was found in the methanolic leaf extract of the micropropagated plantlets (0.96) followed by the methanolic stem extract of micropropagated plant (0.87), while the remaining extracts reduced a lesser amount of Fe^3 +^ to Fe^2 +^. Both DPPH and FRAP indicate a higher antioxidant activity in leaf samples which may be due to high contents of polyphenol, flavonoids and alkaloids present in them. Natural antioxidants, which are present in fruits, vegetables and medicinal plants, have received much attention and have been studied extensively, since they are effective free radical scavengers and are assumed to be less toxic than synthetic antioxidants. Leaves of *D. nobile* should be acknowledged as promising sources of non-toxic natural antioxidants which could be used for cultivation and for breeding programs.

In conclusion, the present investigations suggest that in *D. nobile* low to moderate dose of TDZ can be used efficiently to induce high frequency proliferation of PLBs. Having both medicinal as well as horticultural importance, the present protocol for *D. nobile* is of much significance being rapid and leading to a large number of clones bypassing the need for auxin–cytokinin PGR complement. Being rich in various secondary metabolites, PLBs in particular have significance in orchid research being important for *Agarobacterium*-mediated transformations. Beneficial genes that attribute commercially important traits such as flower color, flower longevity, and resistance to diseases into various cultivars of this economically important orchid genus can be introduced as well as inter-generic hybrids with other related genera can be produced (Men et al., 2003, Mishiba et al., 2005).

The following are the supplementary data related to this article

Table S1 Effect of different concentrations of TDZ and activated charcoal on rooting response of *D. nobile* after 4 months.

Table S2 Re-establishment of *D. nobile* plantlets after 4 months of hardening.

Table S3 Total phenolics, flavonoids, alkaloids and tannin content in different parts of *D. nobile* with respect to different solvent systems (phenolics: mg GAE/g DW; flavonoids: mg QE/g DW; alkaloids: mg/ATP/g DW; tannin: mg TAE/g DW).

## Figures and Tables

**Table 1:** Effect of TDZ incorporated in MS medium on PLB induction and subsequent organogenesis in the segments of pseudostem explants of *D. nobile* after 8 weeks of culture.

Sl	TDZ treatment (mg/l)	Explant response (%)	No of PLBs/explant	PLB weight (g)	No of shoots differentiated/culture	Shoot length (cm)	Root length (cm)
1.	Control	–	–	–	–	–	–
2.	0.10	69.8 ± 0.63 h	3.70 ± 0.48	0.26 ± 0.0078e	2.6 ± 0.51d	1.28 ± 0.032c	1.29 ± 0.004c
3.	0.50	78.7 ± 0.78 g	5.77 ± 0.49b	0.80 ± 0.0094c	3.5 ± 0.52c	1.48 ± 0.022c	1.24 ± 0.051c
4.	1.0	90.8 ± 0.73d	7.50 ± 0.70a	0.86 ± 0.0094c	4.6 ± 0.51b	1.78 ± 0.015b	2.19 ± 0.166b
5.	1.5	94.1 ± 0.73c	11.60 ± 0.69a	1.16 ± 0.0699b	5.9 ± 0.31 a	1.95 ± 0.064a	3.12 ± 0.078a
6.	2.0	64.8 ± 0.63a	14.70 ± 0.48c	1.32 ± 0.0195a	3.9 ± 0.31 ab	1.86 ± 0.047ab	2.14 ± 0.051b
7.	2.5	59.9 ± 0.56b	12.80 ± 0.34c	0.75 ± 0.0122 cd	3.2 ± 0.42d	0.79 ± 0.076d	1.21 ± 0.056 c
8.	3.0	28.3 ± 0.94e	6.90 ± 0.56d	0.67 ± 0.0084d	–	–	–
9.	3.5	21.5 ± 0.84f	6.20 ± 0.42e	0.29 ± 0.0147e	–	–	0.27 ± 0.013

Values are the mean ± SD. Means followed by the same letter in the column are not significantly different as indicated by Fisher's LSD (p = 0.05).

**Table 2:** Data of RAPD and SCoT primers used in the present study and the extent of polymorphism.

Sl no.	Primer Name	Primer Sequence (5′–3′)	Total no. of bands	No. of mono-morphic bands	No. of poly morphic bands	% of poly-morphism (*P*)	Resolving power (*R_p_*)
*RAPD*							
1.	OPA-11	CAATCGCCGT	6	5	1	16.66	9
2.	OPA-12	TCGGCGATAG	4	3	1	25.00	7.33
3.	OPA-13	CAGCACCCAC	4	4	–	–	8
4.	OPB-1	GTTTCGCTCC	2	1	1	50.00	3.66
5.	OPB-6	TGCTCTGCCC	4	4	–	–	8
6.	OPH-19	CTGACCAGCC	5	5	–	–	10
7.	OPK-4	CCGCCCAAAC	2	2	–	–	4

*SCoT*							
8.	S4	CAACAATGGCTACCACCT	2	2	–	–	4
9.	S5	CAACAATGGCTACCACGA	6	6	–	–	12
10.	S6	CAACAATGGCTACCACGC	5	5	–	–	10
11.	S7	CAACAATGGCTACCACGG	3	3	–	–	6
12.	S9	CAACAATGGCTACCACGT	4	4	–	–	8
13.	S10	CAACAATGGCTACCAGCA	4	4	–	–	8
14.	S11	CAACAATGGCTACCAGCC	4	4	–	–	8
15.	S12	ACGACATGGCGACCAACG	5	5	–	–	10
16.	S17	ACCATGGCTACCACCGAG	4	4	–	–	8
17.	S25	ACCATGGCTACCACCGGG	2	2	–	–	4
18.	S26	ACCATGGCTACCACCGTC	4	3	1	25.00	8
19.	S32	CCATGGCTACCACCGCAC	3	3	–	–	6
20.	S33	CCATGGCTACCACCGCAG	4	4	–	–	8
21.	S34	ACCATGGCTACCACCGCA	5	5	–	–	10
22.	S35	CATGGCTACCACCCGCCC	2	1	1	50.00	10
Total			84	79	5	5.95	

**Table 3:** Comparison of RAPD and SCoT markers, individually as well as collectively.

SL	Name of the approach	No. of primer used	Total bands amplified	Avg. bands/primer	Total no. of poly-morphic bands	% of poly-morphism	Distance range (Jaccard's coefficient)	*PIC*^a^	CC^b^^(r)^	DC^c^^(r^2^)^
1.	RAPD	7	27	3.85	3	11.11		0.92		
2.	SCoT	15	57	3.80	2	3.50	0.97–1.00	0.76	0.51	0.26
3.	RAPD + SCoT	22	84	3.81	5	5.95		0.82		

a Polymorphic information content = *PIC*.

b Correlation coefficient = CC.

c Determination coefficient = DC.
