# Supplementary material for: Genetic stability and phytochemical analysis of the in vitro regenerated plants of Dendrobium nobile Lindl., an endangered medicinal orchid
Source: Meta Gene. 2014 Jul 15;2:489–504. doi: 10.1016/j.mgene.2014.06.003 (PMC4287867; doi:10.1016/j.mgene.2014.06.003)
Supplement: Table S1 — Effect of different concentrations of TDZ and activated charcoal on rooting response of D. nobile after 4 months. [file mmc1.doc]

**Table S1** Effect of different concentrations of TDZ and activated charcoal on rooting response of *D. nobile* after 4 months

| **Treatments** | | **Rooting percentage (%)** | **Average number of**  **roots per explant*** | **Mean length of root (cm)*** |
| --- | --- | --- | --- | --- |
|  | |  |  |  |
| **TDZ**  **(mg/l)** | **Charcoal**  **(%)** |  |  |  |
| 0.5 | - | 29.7± 0.82d | 2.14 ± 0.089e | 1.21± 0.014e |
| 0.5 | 0.25 | 30.7± 1.25d | 2.95 ± 0.158d | 1.78 ± 0.032d |
| 0.5 | 0.30 | 34.8± 0.78cd | 3.85 ± 0.337b | 2.03± 0.094bc |
| 1.0 | - | 37.65± 0.62c | 2.85 ± 0.301d | 2.49± 0.021b |
| 1.0 | 0.25 | 94.2 ± 0.78a | 5.41 ± 0.159a | 3.12 ±0.496a |
| 1.0 | 0.30 | 79.9 ± 1.10b | 4.42 ± 0.091b | 1.96±0.040cd |

*Values are the mean ± SD. Means followed by the same letter in the column are not significantly different as indicated by Fisher’s LSD (P=0.05).
