# Supplementary material for: Genetic stability and phytochemical analysis of the in vitro regenerated plants of Dendrobium nobile Lindl., an endangered medicinal orchid
Source: Meta Gene. 2014 Jul 15;2:489–504. doi: 10.1016/j.mgene.2014.06.003 (PMC4287867; doi:10.1016/j.mgene.2014.06.003)
Supplement: Table S2 — Re-establishment of D. nobile plantlets after 4 months of hardening. [file mmc2.doc]

**Table S2** Re-establishment of *Dendrobium nobile* plantlets after 4 months of hardening

| Treatment | Survival % | **Height (cm)** |
| --- | --- | --- |
| Brick pieces + Charcoal clumps (1:1) | 63.7 ± 0.58 | 2.58 ± 0.05 |
| Brick pieces + Charcoal clumps (1:1) + layer of moss | 84.3 ± 0.69 | 3.80 ± 0.11 |
| Brick pieces + Charcoal clumps + Decaying litter (1:1:1) | 41.3 ± 0.52 | 3.63 ± 0.23 |
| Brick pieces + Charcoal clumps + Decaying litter (1:1:1) + layer of moss | 42.2 ± 0.28 | 3.29 ± 0.12 |

Values represent means ± S.D.

Means of 10 values taken and the experiment was repeated thrice.
