# Supplementary material for: Genetic stability and phytochemical analysis of the in vitro regenerated plants of Dendrobium nobile Lindl., an endangered medicinal orchid
Source: Meta Gene. 2014 Jul 15;2:489–504. doi: 10.1016/j.mgene.2014.06.003 (PMC4287867; doi:10.1016/j.mgene.2014.06.003)
Supplement: Table S3 — Total phenolics, flavonoids, alkaloids and tannin content in different parts of D. nobile with respect to different solvent systems (phenolics: mg GAE/g DW; flavonoids: mg QE/g DW; alkaloids: mg/ATP/g DW; tannin: mg TAE/g DW). [file mmc3.doc]

**Table S3** Total phenolics, flavonoids , alkaloids and tannin content in different parts of *D. nobile* with respect to different solvent systems (phenolics: mg GAE/g DW; flavonoids: mg QE/g DW; alkaloids: mg/ ATP/g DW; tannin: mg TAE/g DW

| **Plant Parts** | **Solvent** | **Mother Plant** | | | | **Micropropagated Plant** | | | |
| --- | --- | --- | --- | --- | --- | --- | --- | --- | --- |
| **Stem** | **Methanol**  **Acetone**  **Chloroform** | **TPC** | **TFC** | **TAC** | **TTC** | **TPC** | **TFC** | **TAC** | **TTC** |
|  |  | 38.52±0.15 | 3.23± 0.13 | 34.76±0.32 | 20.55±0.43 | 41.39 ± 0.10 | 5.38±0.12 | 36.23±0.22 | 23.22 ±0.30 |
|  |  | 6.39± 0.17 | 0.99± 0.15 | 22.23±0.44 | 7.29±0.34 | 7.23±0.33 | 0.81±0.32 | 18.39±0.34 | 7.22±0.22 |
|  |  | 5.83± 0.23 | 0.53 ± 0.12 | 20.28±0.43 | 6.13±0.39 | 4.23±0.21 | 0.56±0.23 | 18.28±0.43 | 5.26±0.18 |
| **Leaves** | **Methanol**  **Acetone**  **Chloroform** | 20.53±0.35 | 8.03± 0.54 | 44.76±0.23 | 11.48±0.45 | 25.32±0.15 | 14.39± 0.30 | 54.34±0.19 | 18.38±0.23 |
|  |  | 9.39± 0.43 | 2.59± 0.39 | 31.23±0.25 | 5.36±0.54 | 8.82±0.17 | 2.23±0.34 | 28.39±0.17 | 6.11±0.43 |
|  |  | 3.25± 0.20 | 2.11± 0.44 | 29.88± 0.39 | 3.11± 0.23 | 4.21±0.23 | 2.29±0.43 | 24.56±0.27 | 4.29±0.76 |

GAE Gallic Acid Equivalent; QE Quercetin Equivalent; ATP Atropine Equivelent; TAE Tannic Acid Equivelent; DW Dry Weight; TPC Total Phenolic Content; TFC Total Flavonoid Content; TAC Total Alkaloid Content; TTC Total Tannic Acid Content; Values represent mean ± SE of five replicates.
